# Lectures on Physiology, Zoology, and the Natural History of Man, Delivered at the Royal College of Surgeons

**Published:** 1831

**Authors:** 


Art. VII. *Lectures on Physiology, Zoology, and the Natural History of Man*, delivered at the Royal College of Surgeons—By W. Lawrence, F. R. S., Professor of Anatomy and Surgery to the College, Assistant Surgeon to St. Bartholomew's Hospital, Surgeon to Bridewell and Bethlem Hospitals, and to the London Infirmary for Diseases of the Eye.

The science of Natural History appears to be the peculiar province of the medical profession. Every department of it has been investigated by physicians with peculiar industry and zeal; and if they have not laid the foundations of the edifice, they have completed the structure, and determined its symmetry, order and arrangement. Every subject in any way connected with the profession of medicine they have studied with unremitting ardor, making it their pride, like the ancient Suevi, to conquer every territory bounding on their domain, not, however, that barren deserts may bear testimony to their valor, but that cultivated regions, appropriated to the general interests of mankind, may evince their civilization and humanity.

Of the different provinces of Natural History, none is connected with the science of medicine by more interesting and important relations than zoology. The pharmaceutist has been led to prosecute the study from the number of valuable remedial agents drawn from the animal kingdom. The physiologist and anatomist have pursued the science with more ardor and additional interest, from the light which a knowledge of the structure and physiology of the animal kingdom is calculated to throw upon their own favorite pursuits. A third class, forgetting the primary object of their pursuit, have pursued the collateral science as a principal, and degrading man from his high superiority, have considered him only as a subdivision of one of the classes of animals which inhabit our globe.

To rescue man from this degraded rank in the animal creation, and to assign him a station more agreeable to his vanity, and more consistent with his general pretensions, is, in part, the design of the work before us.

It is not a little curious, however, to observe, that while Dr. L. manifests a very becoming zeal for the nobility of the human family, he, like most persons who affect a great deal of neutrality, manifests a spirit of bitter hostility towards those who, more orthodox than himself, take the liberty of denouncing the investigation of tenets which they consider necessary to the perfection of their system.

Dr. L. contends for the common origin of the human family, and their superiority to the animal creation; but he indulges the bitterest sarcasm towards those who think they are distinguished from the rest of the animal creation by an immaterial spirit. That the author has seen proper to treat the illiberality of his opponent in this manner, we think is very much to be regretted. The fact is now universally admitted, not only that ridicule is a most imperfect test of truth, but that sarcasm is, of all other arguments, the least calculated to convince an opponent. The question is in our opinion entirely beyond the reach of human reason, and the dogmatism of the disputants tends to confirm us in this opinion. Nor ought the decision of this question to have any bearing upon our opinions respecting the immortality of the soul. The belief in this latter doctrine is found so universally to prevail among mankind, of every age and nation, and through every rank and condition of society, that we cannot avoid considering it as one of the principles of the human mind, either immediately implanted by the creator, or made to arise so inevitably from the circumstances in which man is placed in society, as to be considered the work of the author of our existence; and it is this divine sanction to the belief that forms the strongest argument which human reason can offer in its behalf.

But whatever influence the doctrine of materialism ought legitimately to have over our moral sentiments, we are confident that its actual influence has been very injurious. While we admit therefore, that the friend of Truth should boldly follow wherever she leads him, it is by no means to be inferred that every ingenious speculator who disturbs the order of society by opinions which derive their plausibility from the weakness and ignorance of the human mind, should justify his rashness, under the specious show of a disinterested love of truth. With these remarks, we shall proceed to that part of the work in which we are now immediately interested.

This part of the work Mr. L. has divided into two sections—the first, *treating on the difference (corporeal and mental) between man and other animals*—or, in other words, *on the specific character of man:* the second will contain *an enumeration and discussion of the characters by which the different races of mankind are distinguished from each other, and a full consideration of the question whether they ought to be regarded as originally distinct species, or as varieties of a single species.*

Linneus in placing man in the order *primates*, of the class MAMMALIA, in company with the monkeys and bats, has remarked, that he can establish no characteristic by which man can be distinguished from the monkey. Mr. L. very well remarks, that if this were true, Zoology would not deserve the rank of a science.

Man is placed in a separate order with the following zoological distinctions.

Order bimanum (two handed); genus homo; *characters*: erect stature; two hands; teeth approximated and of equal length; the inferior incisors perpendicular; prominent chin; rational; endowed with speech; unarmed; defenceless.

The following summary, introduced by the author as a recapitulation of the charcters of man, will afford a comprehensive view of the proofs of his constituting a separate and distinct species.

1. Smoothness of the skin, and want of natural offensive weapons, or means of defence.

2. Erect stature; to which the conformation of the body in general, and that of the pelvis, lower limbs, and their muscles in particular are accommodated.
3. Incurvation of the Sacrum and os coccygis; and consequent direction of the vagina and urethra forwards.
4. Articulation of the head with the spinal column by the middle of its basis, and want of ligamentum nuchæ.
5. Possession of two hands, and very perfect structure of the hand.
6. Great proportion of the cranium (cerebral cavity) to the face (receptacles of the senses and organs of mastication.)
7. Shortness of the lower jaw, and prominence of its mental portion.
8. Want of the intermaxillary bone.
9. Teeth all of equal length, and approximated: inferior incisors perpendicular.
10. Great developement of the cerebral hemispheres.
11. Great mass of brain, in proportion to the size of the nerves connected with it.
12. Greater number and development of mental faculties, whether intellectual or moral.
13. Speech.
14. Capability of inhabiting all climates and situations; and of living on all kinds of food.
15. Slow growth; long infancy; late puberty.
16. Menstruation; exercise of the sexual functions not confined to particular seasons.

The erect attitude and biped progression are characteristic of man, and adapted to these we have the conformation of the lower limbs; the form and position of the pelvis; the arrangement of the spine; the junction of the head with the vertebral column; the direction of the face and eyes; and the superior extremities formed for prehension and not for the support of the body.

The lower limbs in man are characterized by their length and strength; by the strength of their muscles, and especially of the glutei and gastrocnemii muscles, which have been noticed by the earliest naturalists, as peculiar to man; by their axes coinciding with the centre of gravity of the body; and by the freedom and extent of their motions upon the pelvis.

The peculiarities of the feet are not less distinctly marked, designating them as the sole ultimate support of the body. The whole surface of the tarsus, metatarsus, and toes, and the great protuberance of the os calcis resting on the ground, give them a degree of firmness, solidity and strength, in proportion to the size of the body, that is not possessed by any other animal; and this latter bone forming a right angle with the leg, gives to the powerful muscles which are inserted into it, peculiar facility for supporting the erect attitude.

In all these particulars we observe a striking contrast between man and those animals which most nearly resemble him. Even in the monkey tribes the cervix femoris is short, and in other animals it is entirely wanting, so that the lateral motion of the legs is very much restricted; the thigh bone forms an angle with the spine, and generally an acute one; it is short, straight and nearly concealed by the muscles which cover it; and the leg forms an angle with the thigh, and is kept habitually bent by the muscles inserted into it; the os calcis is raised so as to form an angle with the leg, so that the digitated quadrupeds support themselves on the toes, while in others, this bone is raised perpendicularly, and the extremity of the phalanges rests upon the ground.

The peculiarities of the pelvis do not afford less decisive evidence that the erect position is natural to man.

The peculiarities of the human pelvis coincide with those of the lower limbs. The form of this part is very characteristic in man, and distinguishes him from the simiæ, and indeed from all other mammalia. It might be asserted, that the human skeleton alone has a proper pelvis; that is, such an incurvation of the sacrum and coccyx, and such an union of them with the ossa innominata, as forms a basin-like cavity, from which, the space included between the elongated ilia, and the straight sacrum and coccyx of monkeys, differs toto cœlo. In the orang-outang, and the elephant, we find the nearest approach to the human formation. In the former, however, the upper part of the ilium is narrow and elongated, stretching upwards in the direction of the spine, and its length exceeds its breadth, so that the relations of these two dimensions are very different in man and this animal. In the latter, the symphysis pubis is very deep; and in both, there is neither that incurvation of the sacrum, from the promontory downwards, nor that direction of the coccyx forwards, which, with the broad horizontal expansion of the ilia, and the shallowness of the symphysis pubis, are peculiar to the human frame, and make it a broad and firm basis for the trunk, on which the weight of the abdominal contents, and particularly of the pregnant uterus, is supported. The lower part of the sacrum and the os coccygis are turned forwards in man, and form the only firm bony resistance, in the inferior aperture of the pelvis, to the abdominal viscera, forced downwards by the diaphragm and abdominal muscles. These bones are straight in all other animals, because the weight of the viscera is differently supported. Even in the orang-outang, the sacrum is flat and contracted, and continued, together with the os coccygis, in a straight line with the vertebral column. If the human sacrum and coccyx had been continued in a straight line with the spine, as those of the orang-outang and monkeys are, the ossa innominata remaining as at present, they would have projected beyond those bones, so as to disable us from sitting. The curve which they describe, in man only, obviates this inconvenience; and allows the pelvis to rest securely, in the sitting attitude, on the broad and strong ischiatic tuberosities.

The distribution, size, and offices of the muscles, are also peculiarly appropriate to the erect position. The large glutei muscles forming the buttocks, the exterior of which the largest in the body, preserves the body in the erect position, and the two others supporting the body upon one leg while the other is elevated—are peculiar to man. The extensors of the leg are very powerful in man, to serve the two-fold purpose of supporting the weight of the body, and of carrying the leg forward on the thigh in progression. In animals, on the contrary, the flexors predominate.

The muscles of the ankle joint, and especially those which form the calf, sustain the weight of the body, and are the chief instruments in progression, and from the great weights imposed upon them, are necessarily very powerful, the protuberance therefore, which they form upon the back part of the leg is peculiar to man, and may be considered as a requisite of the erect position.

The whole arrangement of the thorax corresponds to the erect attitude of man, its lateral diameter is very considerable, thereby placing the arms farther apart, and giving greater extent to their motions; it is pyramidical in its shape, thereby lowering the centre of gravity of the body; and since the weight of the abdominal viscera is sustained by the pelvis, its length is contracted by shortening the sternum and reducing the number of the ribs.

In animals, on the other hand, it is compressed laterally but deep, from the sternum to the spine, that the fore legs may be brought nearly together, for more conveniently sustaining the body. Their sternum is long and narrow, and together with the ribs, approaches more nearly to the pelvis, for better supporting the abdominal viscera.

For some peculiarities in the spine and the pelvis, we shall refer to the author.

The spine of man presents some important peculiarities resulting from his characteristic attitude. One of these is, its very remarkable increase of size in the lumbar region ; an augmentation corresponding to that of the superincumbent weight, and to the magnitude of the efforts which this part has to sustain. The immense bulk of the sacrum, far exceeding in proportion to the rest of the body, that of any animal, is referable to the same cause, to the mode in which this weight is transmitted to the hip-bones, and thence to the lower limbs, to the peculiar construction of the pelvis. The waving line of the column, arising from a series of curves in opposite directions, is altogether peculiar to man ; it allows a proper distribution of the weight with respect to the centre of gravity, the line of which, carried through the entire trunk, must fall within the space covered by the feet, or by one foot, when we support the body on one only. As this line passes through all the curves, motion is allowed in the upper regions without impairing the general equilibrium.

I have explained how the lower extremities afford a sufficient base of support and solid columns to sustain the trunk, and how the same point is secured by the organic arrangement of the latter. The breadth of the human pelvis forms an ample basis for the body, and a firm point of action for the abdominal and other muscles, enabling them quickly to rectify the position of the parts above. In all the digitated animals the pelvis is so narrow that the trunk resembles an inverted pyramid : there would be great difficulty in maintaining it in equilibrio, even were it possible for the animal to assume the erect position. In those instances where the pelvis is broader, as in the hoofed animals, the other conditions of the upright stature are absent. The bear, however, forms an exception to these observations, and may be taught to stand and walk erect, although the posture is manifestly irksome to the animal. When quadrupeds endeavor to support themselves on the hind extremities, as for the purpose of seizing any object with the fore feet, they rather sit down than assume the erect position ; for they rest on the thighs, as well as on the feet; and this can only be done where the fore part of the body is small, as in the simiæ, squirrel, &c. In other cases the animal is obliged to support itself by the fore feet also, as in the dog, cat, &c.

It has been already remarked that man is a *two handed* animal, and his hands are so admirably and peculiarly constructed for the purpose of prehension, that the name has been appropriated to his members.

There is a general resemblance of form throughout the upper and lower extremities, in their principal divisions, the number and form of their bones, the structure of their articulations, and in the muscles which are attached to them. This coincidence is so great that the contrast between them cannot fail to impress upon the most casual observer, the different offices which are appropriated to each. Solidity is the characteristic of the latter, and mobility of the former, and such is the modification in the structure and component parts of each, to secure these objects, that the ultimate design is immediately manifest. The lower extremities possess only so much mobility as will enable them to accommodate themselves to the changing positions of the body, and to resist the shock to which it is necessarily exposed by these changes. In the upper extremities every thing is sacrificed to freedom of motion, and owe much of their security to that flexibility of structure that enables them to elude the violence which their more delicate organization could not resist.

In no part is this contrast more striking than in the hands and feet. The meta-carpus and fingers form the principal part of the hand, in which every joint possesses a freedom of articulation that fits it for the nicest manifestations, while the thumb is placed as an opposable member, with a peculiar set of muscles to second its operations.

In the foot on the other hand, the large bones of the tarsus, united by strong articulations, admitting of little motion, form the principal part, and sustain the weight of the body when at rest in the erect position. The metatarsal bone of the great toe, united to the tarsus by a firm articulation, with just sufficient flexibility to impart the elasticity which its situation and office require, is placed parallel with the rest, is moved by the same, or a corresponding set of muscles, and, with them, sustains the body when raised upon the toes, and in walking, leaping, &c.

In comparing this part of the human structure with that of other animals, Mr. L. has the following remarks.

The human hands being terminated by long and flexible members, of which only a small portion is covered by the flat nails, while the rest is furnished with a highly organized and very sensible integument, form admirable organs of touch and instruments of prehension. The animal kingdom exhibits no corresponding part so advantageously constructed in these respects. At the same time, the lateral attachment of the arms to the trunk, and the erect attitude, give us the freest use of these admirable instruments.

Several mammalia have also hands, but much less complete, and less serviceable than that of the human subject, which, in comparison to them, was justly enough termed by the Stagyrite, the organ of all organs. The great superiority of the human hand arises from the size and strength of the thumb, which can be brought into a state of opposition to the fingers, and is hence of the greatest use, in enabling us to grasp spherical bodies, and take up any object in the hand, in giving a firm hold on whatever we seize, in executing all the mechanical processes of the arts, in writing, drawing, cutting: in short, in a thousand offices, which occur every moment of our lives, and which either could not be accomplished at all, if the thumb were absent, or would require the concurrence of both hands, instead of being done by one only.—Hence it has been justly described by Albinus as a second hand, "manus parva majori adjutrix."

All the simiæ possess hands; but the most distinguishing part, the thumb, is slender, short, and weak, even in the most anthropomorphous; regarded as an imitation of the human structure, it would almost justify the term applied to it by Eustachius—"ridiculous." The other fingers are elongated and slender.

The comparison which I have already drawn between the construction of the hand and foot, having shown that the latter is merely calculated for support in man, we may state that he is two-handed and two-footed, or bimanous and biped.

Monkeys, apes, and other anthropomorphous animals, can, in fact, be called neither bipeds nor quadrupeds; but they are quadrumanous, or four-handed. They have opposable thumbs on the lower as well as upper extremities; and thus their feet are instruments of prehension as well as their hands.

We may now answer the question, whether the orang-outang and other simiæ go erect, or on all-fours; they do neither, but live chiefly in trees, for which they are admirably adapted by having prehensible members, instruments for grasping and holding, on both upper and lower extremities. Hence Cuvier calls them "les grimpeurs par excellence." They live in trees, and find their food in them: they can hang by one fore or hind leg, employing the remaining members in gathering fruit, or in other offices. Those which have less perfect hands are furnished with prehensive tails, by which they can be more securely supported in trees.

It is well remarked by the author, that when we see monkeys walking erect, it must be ascribed to instruction and discipline. But as some persons have been found to contend that man ought to go upon all fours, so there are others who undertake to prove that the orang-outang, and the monkey tribe in general, have an organization suited to biped progression. In reply to these the author cites the testimony of the most respectable naturalists to the contrary; but, deeming the evidence drawn from anatomy sufficiently satisfactory, we think it unnecessary to refer to them.

In Chapter IV. Mr. L. introduces the comparison of the human head and teeth to those of other animals, with the following remarks:

When we consider that the head affords a receptacle for the organ of the mind, that it lodges the principal external senses, as well as the instruments for the procuring, receiving, masticating, and swallowing the food, and a considerable part of the apparatus employed in producing sound, we shall not be surprised at the striking differences in its construction, at those proportional developements or contractions of its several parts, which determine the faculties and endowments of different animals, and their relative rank in the scale of nature. The most convenient position for this important assemblage of organs—including the chief means by which we are connected, actively or passively, with the external world—must exhibit corresponding varieties. A situation is required, combining firmness of support with freedom of motion, a ready communication of the senses with their appropriate external objects, and a corresponding arrangement of the entrances to the respiratory, digestive and vocal cavities. The mode in which the entire mass is articulated and supported must therefore be varied according to the predominance or contraction of the various particular organs, as well as in conformity to the attitude of the animal, and the distribution of other parts, particularly the upper limbs. As the proportions of its parts in the human subject indicate a predominance of the organ of thought and reflection over the instruments employed in external sensation, and the supply of merely animal wants, which places man at the top of the intellectual scale; so the position of the whole, and the arrangements for its support and motion, are calculated, like all the details of organization hitherto examined, in reference to his peculiar distinction of the erect attitude.

A very striking difference between man and all other animals consists in the relative proportions of the cranium and face, which are indicated in a general, but not very accurate manner, by the facial line.

One of the most simple modes of expressing the relative proportion of these parts is by the direction of the *facial line*, and the amount of the facial angle. Supposing a skull to be at rest on the occipital condyles, and neither inclining backwards nor forward, a line drawn from the greatest projection of the forehead to that of the upper maxillary bone, is called the *facial line*; and the angle formed by the intersection of this line by a horizontal line, is called the *facial angle*. In man the face is placed under the forehead, and consequently, the facial line will be nearly perpendicular, and the facial angle will approach a right angle: as we recede from man, among the lower orders of animals, the prominence of the forehead diminishes, and that of the jaws increases; consequently, the size of this angle will give a general idea of their relative developement.

In man, the facial angle varies from 65 to 85 in the adult, but the ancient Greeks aware of the intellectual character which a prominent forehead gave to the countenance, in their statues have extended it to 90, and even to 100.

A vertical section of the head, in the longitudinal direction, shews more completely the relative proportions of the face and cranium.

The human and brute face are not more strongly contrasted in size, and in their relation to the cranium, than in their general configuration, and the uses to which they are subservient. The former is an organ of expression, an index to the varying passions and emotions of the mind, imparting a more accurate idea of the moral feelings and character than words can convey. The latter is devoted to merely animal purposes, an instrument for procuring and masticating the food, and often a weapon of offence and defence.

The want of the intermaxillary bone has been assigned by Camper, as one of the characteristics of the human head. The superior maxillary bones of the human subject are united together, and contain the whole of the teeth; but in the mammalia they are separated by a third bone, containing the incisor teeth, and which was called os incisivum. Since, however, it is found in animals which have no incisor teeth, Blumenbach has, more appropriately, called it os intermaxillare. This bone is wanting only in a few of the most anthropomorphous monkeys.

Another distinction between man and animals is found in the articulation of the head with the spine. As the peculiarities of the human body require a very limited extent of motion in the head; the articulation alluded to is placed very nearly in the centre of its base—an arrangement which very much facilitates its support in the erect position. In animals, on the other hand, whose jaws are almost universally used as instruments of prehension, this articulation is placed very nearly or quite at the back part of the head, so as to admit of the greatest possible extent of motion.

The teeth of man are distinguished by being all of one length, and by being arranged in a uniform and unbroken series, and by the inferior incisors being perpendicular, and placed in the same vertical line with the front of the jaw. The obtuse tubercles of the grinders also are peculiar, neither resembling the flat crowns of the herbivorous animals, nor the cutting and tearing grinders of the carnivorous animals; but partaking of the nature of both, and therefore adapted to that mixed diet to which an unerring guide prompts him to resort.

The chin is peculiar to man, and exists as a necessary consequence of the inferior incisors being perpendicular.

In chapter V, Mr. L. in remarking upon the differences in stature and proportion, between man and the simiæ, presents the following strong points of contrast.

The great length of the upper limbs, the predominance of the fore-arm over the upper-arm, the shortness of the lower limbs, and the great length of the hands and feet, are other striking characters of the monkey kind.

The span of the extended arms in man equals the height of the body: it is nearly double that measure in the anthropo-morphous monkeys.—Our upper arm is longer than the fore-arm by two or three inches; in the last mentioned animals, the fore-arm is the longest. In us, the hip-joint divides the body equally; the lower extremity is less than half the height of the body in monkeys. The proportion of the hand and foot to the body is much greater in them than in us; the excess arising from increase in the length of the phalanges. That all these circumstances are very suitable to the climbing habits of the monkey-race, is too obvious to require particular elucidation.

In a monkey of two feet two inches, the humerus measured four and a quarter, the ulna five inches.

The upper extremities of the pongo of Borneo, reach to the ankles, when the animal is erect: its ulna, in the College Museum, is 15 3-4 inches long; the whole height certainly not exceeding five feet. The man, whose gigantic skeleton is preserved in the same place, was eight feet four inches: the ulna, however, is only 13 3-4 inches.

The upper limbs of the gibbon touch the ground when the animal is erect.

The smoothness and nakedness of the human skin furnishes another ground of distinction, "and this circumstance," says the author, "with the absence of all fur, spines and bristles, &c., and the want of those natural offensive weapons, fangs, talons, claws, &c., justify us in denominating the human body as naturally unarmed and defenceless. The deficiency is amply made up by the internal faculties, and the arts to which they give rise."

The following account of some minor points of difference are worthy of notice.

In comparing man with the anthropo-morphous simiæ, it must be noticed further, that one species (satyrus) has no nail on the thumb of the hind hand; and the other, (troglodytes,) according to Tyson, has thirteen ribs. Both of them have a sacrum composed of three pieces only, instead of five, as in the human subject. One at least (satyrus) has one or two large membranous pouches on the front of the neck, under the platysma myoides, communicating with the cavity of the larynx, between the os hyoides and thyroid cartilage, and capable of distention and evacuation at the will of the animal. It has no ligamentum teres in the hip-joint. It has a membranous canal running along the spermatic cord from the abdomen to the tunica viginalis as other monkeys and quadrupeds have; but this does not exist in the chimpanse. The roof of the mouth is nearly black.

I venture to assert that the differences only which have been just enumerated, without any others, would be amply sufficient to establish the dictinction of species; that no example can be adduced of animals deviating so far from the original model of their structure as to exhibit varieties like those just enumerated; and consequently, that the differences in question can be accounted for only by referring the animals to species originally distinct.

There are some points in which man has been erroneously supposed to differ from animals. The approximation of the two eyes is not peculiar; they are much nearer together in the simia.

Many other mammalia, particularly among the quadrumana, have cilia in both eyelids; this is the case in the elephant.

Although the prominent nose, is a striking character of the human face, particularly in comparison with the monkeys, whose very name (simia, from simus) is derived from the flatness of this part, there is a species considerably surpassing man in the length of this feature;—the long-nosed monkey, s. rostrata, or nasalis.

The external ears are not incapable of motion in all men; nor are they moveable in all other mammalia: in the ant-eaters, for example.

Many quadrumana have an organ of touch, and uvula, as well as man.

Again there are some parts, which man alone, or with a few other mammalia, does not possess. Most of these, which are found chiefly in the domesticated kinds, were formerly attributed to man, when human dissections, from want of opportunities, were uncommon.

The paniculus carnosus, or thin subcutaneous stratum of muscular fibres, covering the ventral and lateral parts of the trunk immediately under the skin,—described by Galen and his followers, and even by Vesalius, the great restorer of anatomy and exposer of Galen's errors, as a part of the human body,—does not exist in man, nor, according to Tyson, in the chimpanse. It is found in the monkeys.

The rete mirabile of the cerebral arteries, included by Galen among the parts of the human body, was shown by Vesalius, not to belong to the human structure.

The seventh or suspensory muscle of the eyeball, which is found in the four-footed mammalia, is not seen in man, as Fallopius observed; neither is the alantois or membrana nictitans.

The brain of man forms a remarkable point of contrast between him and other animals. With the exception of the elephant, his brain is much larger than that of any other terraqueous animal. A mode of comparison, however, evidently more satisfactory than that derived from the absolute weight of the brain, is to be found in the ratio which this viscus bears to the body. Modern physiologists, however, have been very much perplexed by finding that several of the mammalia equal man in this mode of comparison, and that some of the small birds even exceed him. To remove this difficulty, Soemmering has suggested a new point of comparison, viz. that of the ratio which the mass of the brain bears to the bulk of the nerves arising from it. Thus if we divide the brain into two parts, the first of which being connected with the nerves, is devoted to those wants and purposes which we have in common with the animals; the second will include the part of the brain which may be considered as the seat of the mental phenomena.

In proportion, then, as any animal possesses a larger share of the latter and more noble part,—that is, in proportion as the organ of reflection exceeds that of the external senses,—may we expect to find the powers of the mind more diversified and more fully developed. In this point of view man is decidedly pre-eminent; although in his senses and common animal properties, he holds only a middle rank, here he surpasses all other animals that have been hitherto investigated: he is the first of living beings. "All the simiæ," says this accomplished anatomist, "for I have been fortunate enough to procure specimens of the four principal divisions, come after him; for although the proportion of their brain to the body, particularly in the small species with prehensile tails, is equal to that of man, their very large eyes, ears, tongue, and jaws, require a much larger mass of brain than the corresponding parts in the human subject; and if you remove this, the ratio of the brain to the body is much diminished.

"Animals of various kinds seem to me to possess a larger or smaller quantity of this superabundant portion of brain, according to the degree of their sagacity and docility. The largest brain of a horse, which I possess, weighs one pound seven ounces; the smallest human brain that I have met with in an adult, two pounds five ounces and a quarter.[1] But the nerves in the basis of the horse's brain are ten times larger than in the other instance, although it weighs less by fourteen ounces and a quarter.

"But we are not hastily to conclude that the human species have smaller nerves than any other animals. In order that my ideas may be better understood, I shall state the following imaginary case.—Suppose the ball of the eye to require 600 nervous fibrils in one instance; and in another, half the size, 300: further, that the animal with 600 fibrils possesses a brain of seven, and that with 300 a brain of only five drams. To the latter we ought to ascribe the larger brain, and a more ample capacity of registering the impressions made on the organ of vision. For, allowing one dram of encephalon to 100 fibrils, the brain, which is absolutely the least, will have an overplus of two drams, while the larger has only one. That the eye, which is supplied with a double quantity of fibrils, may be a more perfect organ of sense, will be readily admitted; but that point is not connected with the present question."

Upon these different views Mr. L. offers the following remarks:

The basis of the position so much insisted on by Soemmering, is an assumption that a certain bulk of nerve requires always the same proportion of brain for the execution of its office—a datum by no means self-evident. The comparison of the nerves to the *brain* in general is not satisfactory; we should wish to know the relative proportions of the cerebrum, cerebellum, and medulla oblongata. The latter, in deed, is an important point; as most of the nerves are immediately connected with it, few with the cerebrum, and none with the cerebel lum, properly so called.

The most striking character of the human brain is the prodigious developement of the cerebral hemispheres, to which no animal, whatever ratio its whole encephalon may bear to its body, affords any parallel.

It is also the most perfect in the number and developement of its parts; none being found in any animal, which man has not; while several of those found in man are either reduced in size, or deficient in various animals. Hence it has been said, that by taking away, di minishing, or changing proportions, you might form, from the human brain, that of any animal; while, on the contrary, there is none from which you could in like manner construct the brain of man.

It approaches the most nearly to the spherical figure. That the nerves are the smallest in proportion to the brain, has been already pointed out: the brain diminishes, and the nerves increase from man downwards. In the fetus and child, the nerves are proportionally larger than in the adult.

The assertion that it has the largest cerebrum in proportion to the cerebellum does not seem to be quite correct. It has, however, the largest cerebrum in proportion to the medulla oblongata and spinalis, with the single and indeed singular exception of the dolphin.

It has the deepest and most numerous convolutions, apparently in consequence of its size, as the purpose of this structure seems to be that of affording a more extensive surface for the application of the vascular membrane, the pia mater. The convolutions become fewer and shallower as the brain diminishes in size; there are none in the rodentia; none in very small brains.

It has the greatest quantity of medullary substance in proportion to the cortical. In the fetus, the cortical is much more abundant than in the adult.

The difference in the position of the heart is familiar to all. On some peculiarities of the pelvis the following remarks are worthy of notice.

The curvature of the sacrum and os coccygis gives rise to the peculiar situation and direction of the sexual organs, and particularly of the vagina in the human female. As these bones are extended in the same straight line with the spine in all other mammalia, the canal of the vagina follows the axis of the pelvis, lies nearly parallel to the spine, and has its external orifice directed downwards or backwards: the orifice of the urethra opens into the vagina itself. These arrangements fully explain to us why brutes discharge their urine behind, why they copulate backwards, and why parturition is so easy with them.

In these points of structure the monkey kind agree with the mammalia in general, and differ from man. The axis of the vagina is directed downwards in them; the urine is discharged within it (such at least Blumenbach found to be the case in the papio maimon and the simia cynomolgus,) and they are, consequently, retro-mingent and retro-copulant.

The incurvation of the sacrum and coccyx turns the human vagina forwards, so that its axis cuts that of the pelvis nearly at right angles, and its anterior opening is turned forwards: the urethra opens on its upper and front edge, not at all within the canal. Hence the human female differs from all other mammalia in not being retro-mingent and retro-copulant; hence, too, although many inconveniences to which she would have been otherwise exposed, particularly during pregnancy, are obviated, parturition is rendered much more difficult, and a physical reason is found for that doom under which she labours, of bringing forth children in sorrow and in pain.

Although it cannot be deemed an internal organ, this seems the fittest place for mentioning the hymen, an interesting part of the female structure in many respects, and therefore more noticed and investigated than so small a fold of skin would have seemed to deserve. The general opinion of its non-existence in the other mammalia besides man, and the circumstance of its being found in women only at a particular period of life, and even then not universally, have led many anatomists to deny its existence altogether. The question, however, can be so easily settled by direct evidence, that we are surprised to find Buffon still contesting the point. Though the opinion of this great naturalist is incorrect in point of fact, we cannot but admire the eloquence with which he inveighs against the disgraceful opinions and practices which have prevailed on this subject.

It has been generally asserted that this little part is found only in the human subject. In the female orang-outang, Camper says that the hymen was not apparent, although the individual was very young. Blumenbach informs us that he could neither find any trace of this part, nor those supposed remains of it called carunculæ myrtiformes, in monkeys or baboons; and that his search was equally fruitless in a female elephant, in which it had been reported that a hymen existed. Cuvier, on the contrary, represents that several mammalia have a distinct membranous fold at the entrance of the vagina, and others a decided contraction in the same situation.

The clitoris and the nymphæ have been supposed peculiar to the human female, as well as the hymen; the latter, indeed, are generally absent in the mammalia; but Blumenbach informs us that a lemur, which he kept alive for many years, had them very closely resembling the human. The clitoris seems to be universally found in the mammalia: it is very large in the monkey kind, and in the carnivora; and Blumenbach saw it of the size of a fig in a balæna boops, stranded on the coast of Holland.

Man is not less remarkable for that peculiarity of his economy, which enables him to adapt himself to every variety of situation and clime. "The neighborhood of the pole and the equator, high mountains and deep valleys, are occupied by him; his strong but pliant body bears cold, heat, moisture, light or heavy air; he can thrive any where, and runs into less remarkable varieties than any other animals which occupy so great a diversity of abodes."

The Greenlanders and Eskimaux reach to between 70 and 80 deg. of north lat., a climate in which mercury freezes, and expose themselves to a degree of cold which even the polar bear seems incapable of supporting; and even the natives of temperate climates have sustained the winters of these high latitudes with impunity. On the other hand, men can live in a climate where the thermometer rises to 100, 110, or 115, and have borne with impunity, for a short time, a temperature superior to that of boiling water.

The varieties of climate to which his condition can be accommodated is not greater than the change of diet which these different locations require, to preserve the body in a healthy condition. The intention of nature in providing a rich supply of vegetable food for the inhabitants of tropical regions, is not more obvious than is the necessity for animal food in its most stimulating form, for the inhabitants of high northern latitudes. The Greenlanders drink the blood of the seal when warm, and eat his flesh when raw or half putrid, with the keenest relish; and whale oil as a luxury is sought after with an avidity scarcely less than the more potent stimulus of ardent spirits.

In temperate climates man indulges with impunity in every variety of diet, and although whim, caprice, or superstition has given rise to many opinions upon the subject of the injurious tendency of an exclusive animal or vegetable food, experience does not justify the opinion that it exerts any material influence over the physical, moral, or intellectual faculties.

The following extract is not less beautiful in its style, than correct in its reasoning.

Vegetable diet is as little connected with weakness and cowardice, as that of animal matters is with physical force and courage. That men can be perfectly nourished, and their bodily and mental capabilities be fully developed in any climates by a diet purely vegetable, admits of abundant proof from experience. In the periods of their greatest simplicity, manliness, and bravery, the Greeks and Romans appear to have lived almost entirely on plain vegetable preparations: indifferent bread fruits, and other produce of the earth, are the chief nourishment of the modern Italians, and of the mass of the population in most countries of Europe: of those more immediately known to ourselves, the Irish and Scotch may be mentioned; who are certainly not rendered weaker than their English fellow-subjects by their freer use of vegetable aliment. The negroes, whose great bodily powers are well known, feed chiefly on vegetable substances; and the same is the case with the South-Sea Islanders, whose agility and strength were so great, that the stoutest and most expert English sailors had no chance with them in wrestling and boxing.

The representations of the Pythagoreans respecting the noxious and debilitating effects of animal food, are, on the other hand, the mere offspring of imagination. We have not the shadow of a proof, unless we admit Ovid's Metamorphoses and other poetical compositions, that this state of innocence, of exalted temperance, and of entire abstinence from flesh, of perfect tranquility, of profound peace, ever existed, or that it is more than a fable, designed to convey moral instruction. If the experience of every individual were not sufficient to convince him that the use of animal food is quite consistent with the greatest strength of body and most exalted energy of mind, this truth is proclaimed by the voice of all history. A few hundreds of Europeans hold in bondage the vegetable-eating millions of the East. If the Romans, in their earliest state, employed a simple vegetable diet, their glorious career went on uninterruptedly after they had become more carnivorous: we see them winding their way, from a beginning so inconsiderable that it is lost in the obscurity of fable, to the empire of the world: we see them, by the power of intellect, establishing that dominion, which they had acquired by the sword, and producing such compositions in poetry, oratory, philosophy, and history, as are at once the admiration and despair of succeeding ages: we see our own countrymen rivalling them in arts and in arms, exhibiting no less signal bravery in the field and on the ocean; and displaying in a Milton and Shakspeare, in a Newton, Bacon, and Locke, in a Chatham, Erskine, and Fox, no less mental energy. Yet, with these proofs before their eyes, men are actually found who would have us believe on the faith of some insulated, exaggerated, and misrepresented facts, and still more miserable hypotheses, that the developement, form, and powers of the body are impaired and lessened, and the intellectual and moral faculties injured and perverted by animal food.

The comparison of the digestive organs of man to those of other animals, has been supposed to confirm the voice of experience in this respect. Mr. L. however, in the following remarks, seems to take the opposite view of the subject.

The molar teeth being the instruments employed in dividing and preparing the food, must exhibit, in figure and construction, a relation to the nature of the aliment. They rise, in the true carnivora, into sharp-pointed prominences; and those of the lower shut within those of the upper jaw;—when the series is viewed together, the general outline may be compared to the teeth of a saw. These animals are also furnished with long, pointed, and strong cuspidati or canine teeth, which are employed as weapons of offence and defence, and are very serviceable in seizing and lacerating their prey; they constitute in some animals, as the lion, tiger, &c. very formidable weapons. The herbivorous animals are not armed with these terrible canine teeth; their molares have broad flat surfaces, opposed in a vertical line to each other in the two jaws. Plates of enamel are intermixed with the bone of the tooth in the latter; and, as its superior hardness makes it wear less rapidly than the other textures of the teeth, it appears on the grinding surface in rising ridges which must greatly increase the triturating effect. In carnivorous animals the enamel is confined altogether to the surface of the teeth.

The articulation of the lower jaw differs in the two cases as much as the structure of the teeth. In the carnivora it can only move backwards and forwards; all lateral motion being precluded by the rising edges of the glenoid cavity: in the herbivora it has, moreover, motion from side to side. Thus we observe, in the flesh-eaters, teeth calculated only for tearing, subservient, in part at least, to the procuring of food, as well as to purposes of defence; and an articulation of the lower jaw, that precludes all lateral motion. In those which live on vegetables, the form of the teeth and the nature of the joint are calculated for the lateral or grinding motion. The former, having rudely torn and divided the food, swallow it in masses, while in the latter it undergoes considerable comminution before it is swallowed. The teeth of man have not the slightest resemblance to those of the carnivorous animals, except that their enamel is confined to the external surface. He possesses, indeed, teeth called canine, but they do not exceed the level of the others, and are obviously unsuited to the purposes which the corresponding teeth execute in carnivorous animals. The obtuse tubercles of the human molares have not the most remote resemblance to the pointed projections of these teeth in carnivorous animals: they are as clearly distinguished from the flat crowns with intermixed enamel of the herbivorous molares. In the freedom of lateral motion, however, the human inferior maxilla more nearly resembles that of the herbivora.

The teeth and jaws of man are in all respects much more similar to those of monkeys, than of any other animals. A skull, apparently of the orang-outang, in the Museum of the College, has the first set of teeth; the number is the same as in man, and the form so closely similar, that they might easily be mistaken for human. In most other simiæ the canine teeth are much longer and stronger than in us; and so far these animals have a more carnivorous character. The points and ridges of the molares in simiæ are distinguished by their sharpness, from the peculiar obtuse tubercles of the human molares.

The length and divisions of the alimentary canal are very different according to the kind of food. In the proper carnivorous animals, the canal is very short;* the large intestine cylindrical, and the cæcum, not larger than the rest. The form of the stomach and the dispositions of its openings are calculated to allow a quick passage of the food. In

---

*The length of the body, in a straight line from the snout to the anus, compared to that of the intestines, varies in the carnivora, according to Cuvier, from 1: 3 to 1: 5. 8; excepting the hyæna, where it is as 1: 8. 3. "Lec. d'Anat. Comp." iii. 450.

the herbivora, the whole canal is long;* and there is either a complicated stomach, or a very large cæcum, and a sacculated colon: the stomach, even where simple, is so formed as to retain the food for a considerable time.

In comparing the length of the intestines to that of the body, in man and other animals, a difficulty arises on account of the legs, which are included in the measurement of the body in the former, and not in the latter. The great depth of the cranium in man makes a further addition to the length of body, and thereby diminishes the proportion which the intestine bears to it. As our legs are half the height of the body, that should be reduced one half, when it is compared to that of animals measured from the head to the anus; or the length of the intestines may be doubled. When allowance is made for this circumstance, man will be placed nearly on the same line with the monkey race, and will be removed to a considerable distance from the proper carnivora. Soemmering states that the intestinal canal of man varies from three to eight times the length of the body. In Tyson's chimpanse of twenty-six inches, the canal measured one hundred and fifty-nine inches, or about six times the length of the body. In two sapajous and two monkeys, the intestines were respectively 62 and 96 inches; as the body is said in all to have been about 14 inches from the head to the anus, its proportion to the intestines will be in the former as 1: 4 6-14, in the latter as 1: 6 12-14. From these as well as other instances, it is apparent that the comparative length of the alimentary canal in simiæ, is less than in man.

The form of the stomach and cæcum, and the structure of the whole canal, are very much alike in man and the monkey kind. The orangs (S. satyrus, troglodytes, and gibbon) have the appendix vermiformis, which the others want.

Thus we find, that whether we consider the teeth and jaws, or the immediate instruments of digestion, the human structure closely resembles that of the simiæ; all of which, in their natural state, are completely herbivorous.

Man possesses a tolerably large cæcum, and a cellular colon, which, I believe, are not found in any carnivorous animal.

I do not infer from these circumstances that man is designed by nature to feed on vegetables, or that it would be more advantageous to him to adopt that diet. The hands and the arts of man procure for him the food which carnivorous animals earn by their teeth. The processes of cookery bring what he eats into a different state from that in which it is employed either by carnivorous, or herbivorous animals. Hence the analogy in the modes of procuring and preparing food is too loose for us to place much confidence in the results of these comparative views. We must trust to experience alone for elucidating the great problem of diet: its decision has been long ago pronounced, and will hardly now be reversed.

---

*In the ruminantia, the comparative lengths of the body and intestines vary between 1: 11 and 1: 28; in the solipeda, between 1: 8 and 1: 10. Ibid. 453 and 4.

In relation to the power of man to accommodate his constitution to every variety of climate, the question is frequently asked, whether it depends upon physical endowments, upon the strength and flexibility of the human frame, or whether it arises from the protection which human art and contrivance give him against the elements which surround him. In reply to this, it may be remarked, that man evidently derives great advantage from his reason and foresight, but he derives no advantage from those which nature has not bestowed upon many other animals, in giving them instincts as unerring as the dictates of reason. Nature either in their organization, habits, or in the food upon which they subsist, has assigned to all animals limits which they cannot pass. The dog, only, follows his master into every climate, but it requires several generations to produce the requisite changes of bodily structure, and other properties, and when these are effected, the characters of the animal are so modified, that the identity of the species can scarcely be recognized.

The menstrual discharge is peculiar to women. The popular notion which extends this discharge to other animals, will scarcely stand in competition with the opinion of Blumenbach.

"I know, indeed," says Blumenbach, "that the same discharge has been ascribed to other animals, particularly of the order quadrumana. I have carefully inquired about all the female monkeys, which I have seen for these twenty years, either in menageries or carried about for public exhibition, and have found some of them liable to uterine hæmorrhage which observed no period, and was regarded by the more intelligent keepers as a circumstance arising from disease; although they acknowledged, that, in order to excite the admiration of their visitors, they often represent it as true menstruation."

The *intellectual faculties* of man constitute a more important ground of distinction between him and other animals, than any to which we have yet referred. By the aid of these he has reduced the animal kingdom to subjection, and made it tributary to his wants and his luxuries, and supplementary to the defects of his physical organization; he has made himself acquainted with the principle of the most mysterious operations of nature, and divested them of their power to annoy him, or rendered them, in an infinite variety of ways, subservient to his convenience; he embodies the operations of these noble faculties in language, and thus appropriates to each individual the united discoveries of a whole society. They have enabled him to invent a still more wonderful written language, to serve as a medium of communication between distant nations and generations, circulating the benefits of individual discovery, and perpetuating them to become the ground work of successive inventions, until their united contributions have so improved the condition of mankind, as to impose a new face upon their moral and social relations, and to invest those who enjoy these advantages with a character and attributes so superior to the untutored savage, that the identity of the species is scarcely recognized.

Man is not less distinguished by his *social feelings* than by his intellectual faculties. Animals are brought together by *instincts* sufficiently powerful to preserve the bond of union, when necessary for their common safety, or for the reproduction of the species. In addition to this animal impulse, in human society, the social principle is strengthened by innumerable associations, which modify it in such a manner, that the original instinct is almost lost sight of in the amiable sympathies, and ardent feelings, which it is the occasion of producing.

In most of the feelings of which other individuals of the species are the subjects, and in all which come under the denomination of moral sentiments, there is a marked difference between man and animals, and a decided inferiority of the latter. The attachment of the mother to the offspring, so long as its wants and feebleness require her aid and defence, seems as strong in the animal as in the human being, and bears equally in both, the characters of actions termed instinctive. Its duration is confined in the former case, even in social animals, to the period of helplessness; and the animal instinct is not succeeded, as in man, by that continued intercourse of affection and kind offices, and those endearing relations, which constitute the most exalted pleasures of human life.

Of courage, the animal kingdom offers many examples; and the moralists have celebrated the attachment of the dog to his master. It may be doubted whether he can find any instance of such feeling between animals themselves, excepting some cases of sexual unions.

In general, they seem entirely destitute of sympathy with each other, indifferent to each other's sufferings or joys, and unmoved by the worst usage or acutest pangs of their fellows. Indeed, if we except some associated labors in the insect class, principally referring to the continuation of the species, and securing a supply of food, and some joint operations of the male and female in the higher classes, animals seem entirely incapable of concert or co-operation for common purposes, of combining various exertions for the attainment of a common end. This appears to arise from the limited nature and extent of their knowing and reflecting powers; to which, probably, we must refer their incapability of conceiving moral relations.

Laughing and weeping are natural signs in man of certain mental affections, and probably are also peculiar to him; animals are not susceptible of the emotions or states of mind indicated by these external signs.

Man is also distinguished by the moral ties which unite him to his Creator—ties which so invariably exist, in every form of human society, that we must believe their germs to be implanted in the constitution of our minds, by the author of our existence, to spring up, with or without culture, with such a degree of luxuriance, and so entwining themselves with our social and domestic relations, that they can only be eradicated by a long course of crime, or a deliberate perversion of the intellectual faculties. The habitual influence of this *moral principle* constitutes one of the most effectual preservatives of social order, and although its influence may pass unobserved among the numerous principles which combine to modify and regulate the human character, yet, if its salutary restraints were removed, the violence of human passions would convert sosiety into a field of blood. Attempts have been made to throw a doubt over the existence of this important principle, by referring to the terrible excesses which its unenlightened and misguided energies have produced. But would any one doubt the existence of our animal propensities because their unrestrained indulgence produces disease, or can the examples of cruelty and revenge, which result from the partial or perverted operation of our social faculties, justify us in denying that they act at all. Upon the same principle do the cruelties of superstition and bigotry afford additional evidence of a truth universally acknowledged by the "savage and the sage," except where the heart has been corrupted by vicious indulgence, or the judgment, by vain speculation, been led to despise the simple dictates of nature.

It savours as little of sound philosophy to question the existence of a principle because its perfect development and legitimate influence can only be realized by the most assiduous cultivation. The most valuable gifts of God to man are slow in their growth, precarious in their tenure, and followed in their abuse by the most pernicious consequences. The animal feelings which are immediately necessary to individual preservation, appear simultaneous with birth, before the heart can feel, or the judgment comprehend the social relations. But these propensities are but the germs of those powerful instincts which direct the lower animals with so much certainty, to the fulfilment of the objects of their creation, and prove, by the subordinate station which they occupy in man, that he is intended for nobler purposes than to occupy and perpetuate an isolated animal existence.

The sympathetic feelings are to the community, what the animal propensities are to the individual: slower in their growth, and requiring, in order to their full development, a more careful nurture, they evince, when thus cultivated, by their powerful control, the superior importance which Providence attaches to our social relations.

Out of these relations insensibly springs the sense of the obligations which man owes to a superior being—a feeling which requires so much cultivation and so happy a combination of circumstances in order to its full developement, that its pure and perfect influence upon the human character is almost never witnessed; but still evincing, by the necessity of its existence to the preservation of society, the noble destinies which await us on the termination of this transitory existence.

The vagary of *innate ideas* has long since been exploded. We can no more conceive of our moral obligation to God, without some knowledge of his character and attributes, than we can feel the sympathies of a child without having experienced the tenderness of a parent: Yet the absurdity of supposing the existence of attributes without an object, is scarcely excelled by that of the philosopher who questions the existence of a principle, from the facility with which he can trace its origin.

We have been led into this train of remark by the fact, that the author has *cautiously* avoided all allusion to this important principle—an omission of the more consequence, on account of the charge which is often made (we believe unjustly,) of the tendency of our profession to foster principles unfavourable to morality. F.

